# Genetic spectrum and clinical features in a cohort of Chinese patients with autosomal recessive cerebellar ataxias

**DOI:** 10.1186/s40035-021-00264-z

**Published:** 2021-10-18

**Authors:** Hao-Ling Cheng, Ya-Ru Shao, Yi Dong, Hai-Lin Dong, Lu Yang, Yin Ma, Ying Shen, Zhi-Ying Wu

**Affiliations:** 1 Department of Neurology and Research Center of Neurology in Second Affiliated Hospital, and Key Laboratory of Medical Neurobiology of Zhejiang Province, Zhejiang University School of Medicine, Hangzhou, 310000 China; 2 Department of Neurology, Huashan Hospital, Shanghai Medical College, Fudan University, Shanghai, 200000 China; 3 Institute of Neuroscience, Zhejiang University School of Medicine, Hangzhou, 310000 China; 4 CAS Center for Excellence in Brain Science and Intelligence Technology, Shanghai, 200000 China

**Keywords:** Autosomal recessive cerebellar ataxias, Chinese, Genetic spectrum, Structural variation, Clinical features

## Abstract

**Background:**

Although many causative genes have been uncovered in recent years, genetic diagnosis is still missing for approximately 50% of autosomal recessive cerebellar ataxia (ARCA) patients. Few studies have been performed to determine the genetic spectrum and clinical profile of ARCA patients in the Chinese population.

**Methods:**

Fifty-four Chinese index patients with unexplained autosomal recessive or sporadic ataxia were investigated by whole-exome sequencing (WES) and copy number variation (CNV) calling with ExomeDepth. Likely causal CNV predictions were validated by CNVseq.

**Results:**

Thirty-eight mutations including 29 novel ones were identified in 25 out of the 54 patients, providing a 46.3% positive molecular diagnostic rate. Ten different genes were involved, of which four most common genes were *SACS*, *SYNE1*, *ADCK3* and *SETX*, which accounted for 76.0% (19/25) of the positive cases. The de novo microdeletion in *SACS* was reported for the first time in China and the uniparental disomy of *ADCK3* was reported for the first time worldwide. Clinical features of the patients carrying *SACS*, *SYNE1 *and *ADCK3* mutations were summarized.

**Conclusions:**

Our results expand the genetic spectrum and clinical profiles of ARCA patients, demonstrate the high efficiency and reliability of WES combined with CNV analysis in the diagnosis of suspected ARCA, and emphasize the importance of complete bioinformatics analysis of WES data for accurate diagnosis.

**Supplementary Information:**

The online version contains supplementary material available at 10.1186/s40035-021-00264-z.

## Background

Autosomal recessive cerebellar ataxias (ARCAs) are a heterogeneous group of rare metabolic and degenerative genetic disorders that are characterized by progressive damage of the cerebellum and/or its associated afferent tracts [1, 2]. The overall prevalence of ARCAs is estimated to be 3–5 per 100,000 in the general population [3], but each individual type may have varied prevalence among ethnic groups [2, 4]. Most ARCAs have an early onset, occurring before 30 years old, and a major clinical feature of progressive cerebellar ataxia, variably accompanied by dysarthria, ophthalmoplegia, pyramidal and extrapyramidal signs, peripheral neuropathy, cognitive impairment and other symptoms [1, 5, 6].

With advanced technologies in genetic screening, more than 90 genes have been identified in ARCAs [6]. Friedreich’s ataxia (FRDA) is the most common type of ARCA, accounting for up to 25% of all ARCAs, followed by ataxia-telangiectasia or ataxia with oculomotor apraxia (AOA) [1, 2]. It is now increasingly acknowledged that spectrin repeat-containing nuclear envelope protein (*SYNE1*) ataxia and autosomal-recessive spastic ataxia of Charlevoix-Saguenay (ARSACS; gene: *SACS*), which were long thought to be largely confined to a specific group of French-Canadian populations, are relatively frequent ARCAs distributed around the world [2, 7]. Despite the discovery of many disease-causing genes in recent years, the genetic cause of ARCAs remains elusive in more than 50% of affected individuals [2, 8, 9]. The advent of whole-exome sequencing (WES) technology has enabled efficient diagnosis with single-nucleotide variants (SNVs) and small indels [10], and new WES-based methods, such as ExomeDepth, have enabled detection of structural variation like copy number variations (CNVs) in many hereditary diseases [11]. Moreover, due to the low incidence, few studies have reported the genetic or clinical characteristic of ARCA patients in the Chinese population.

In this study, we performed WES and CNV calling for 54 unrelated Chinese patients with autosomal recessive or sporadic hereditary cerebellar ataxia (HCA) to assess the possibility and prevalence of mutations.

## Methods

### Subjects

All patients were consecutively enrolled from Huashan Hospital of Fudan University and the Second Affiliated Hospital of Zhejiang University School of Medicine between August 9, 2008 and May 5, 2021. The patients were evaluated and diagnosed with HCA based on Harding’s criteria [12] by at least two senior neurologists. Informed consent was signed by all participants or their guardians. This study was approved by the Ethics Committees of the above two hospitals.

Fifty-four patients meeting the following inclusion criteria were enrolled in this study: (1) having progressive cerebellar ataxia; (2) being negative for molecular analysis of mitochondrial ataxia or 10 common subtypes of spinocerebellar ataxia (SCA) (including SCA1, 2, 3, 6, 7, 8, 10, 12, 17 and DRPLA) resulting from dynamic mutations or Huntington’s disease; (3) exclusion of other identified etiologies, e.g., multiple system atrophy, Niemann–Pick disease, Parkinson’s disease, Wilson’s disease, multiple sclerosis, viral infection, alcohol or drug intoxication, or paraneoplastic syndrome; (4) autosomal recessive inheritance with the presence of patients in siblings and/or consanguineous union of the parents (*n* = 12); and (5) sporadic cases with onset before the age of 40 years [5, 13] (*n* = 42). In addition, 1000 unrelated Chinese individuals without a history of ataxia were included as the control.

### Genomic DNA extraction and whole-exome sequencing

Genomic DNA was extracted from peripheral blood samples of all patients by using the QIAamp Blood Genome Extraction Kit (Qiagen, Germany) following a standard protocol. Whole-exome Illumina sequencing of DNA was performed according to a detailed protocol described in our previous study [14]. All variants were annotated by ANNOVAR. Two public databases, the 1000 Genomes Project (http://browser.1000genomes.org) and the Exome Aggregation Consortium (http://exac.broadinstitute.org/), and our in-house WES database containing 1000 Chinese control individuals, were used to check the frequency of the variants in the general population. Three software programs, SIFT (http://sift.jcvi.org/), PolyPhen-2 (http://genetics.bwh.harvard.edu/pph2/) and Mutation Taster (http://www.mutationtaster.org/), were used to predict the possible deleterious effects of mutations. Moreover, these variants were compared with the Human Gene Mutation Database (HGMD, Professional 2021.1, http://www.hgmd.cf.ac.uk/) to determine whether they were known or novel. Finally, the interpretation and classification of variants was performed based on the American College of Medical Genetics and Genomics (ACMG) standards [15].

### CNV analysis

CNVs were called from read-depth of WES data using ExomeDepth algorithm according to the developers’ guidelines [16]. For these analyses, each test exome was compared with a set of matched, aggregate reference samples. CNV calls were annotated using AnnotSV [17]. Candidate CNVs were prioritized by exon number, Bayes factor, minor allele frequency and the ratio of observed/expected number of reads. Candidate CNVs were further proved by CNVseq, which performed a low-coverage WGS strategy and included DNA extraction, interruption, library construction and sequencing by Illumina HiSeq 2500 (Illumina, San Diego, CA). The databases ISCA, DGV, Decipher, OMIM, ClinVar and ClinGen were used to analyze the CNVs. Comprehensive assessments of CNV hazard levels were undertaken based on a frequency database and annotation information according to the ACMG standards and guidelines [18].

### Affymetrix CytoScan® Dx assay

The Affymetrix CytoScan® Dx Assay was used to identify the uniparental disomy (UPD), which utilizes a high-density combined SNP and comparative genomic hybridization array platform, which assesses approximately 2,696,550 markers, including approximately 750,000 SNP markers. The whole-genome screening and analysis of chromosomal rearrangements was performed by Affymetrix CytoScan® Dx Assay according to the manufacturer’s recommendations [19].

### Sanger sequencing and parent analysis

After analyzing and filtering the WES data, Sanger sequencing was performed to confirm the sequencing results and the family co-segregation pattern. The parenthood of patients with de novo variants was analyzed using 21 core short tandem repeat regions, including D19S433, D5S818, D21S11, D18S51, D6S1043, AMEL, D3S1358, D13S317, D7S820, D16S539, CSF1PO, Penta D, D2S441, vWA, D8S1179, TPOX, Penta E, TH01, D12S391, D2S1338, and FGA.

## Results

### Twenty-nine novel variants were identified in 25 unrelated ARCA patients

**Fig. 1:**
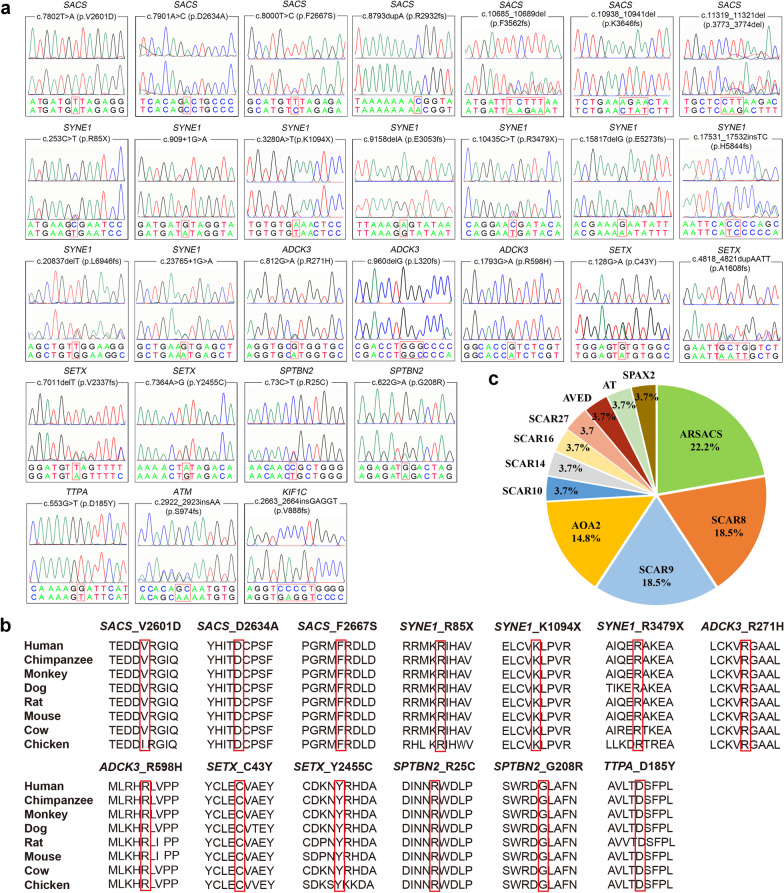
Chromatogram, homology comparison, and genetic spectrum. **a** Chromatograms of 28 novel variants. The upper chromatograms each represent the normal sequence and the lower ones represent the variant. **b** Highlighted zones indicating 13 novel missense variants among 8 species. **c** Distribution of ARCA subtypes with different genetic causes in our ARCA families (*n* = 27, including two previously reported cases)

A total of 38 variants (Table 1) including 29 novel and nine known variants in 10 genes were identified in 25 out of 54 ARCA patients (chromatograms for novel variants except the chr13:23490196-24866656del are shown in Fig. 1a). All novel variants were absent from or present at extremely low frequency in both public databases and our in-house WES database. The pathogenicity of variants was consistently predicted by different in silico prediction programs. All of the novel missense variants were highly conserved among animal species (Fig. 1b). According to the ACMG standard, 17 out of the 29 novel variants were classified as pathogenic variants, 10 as likely pathogenic variants, and the remaining two (*ADCK3*: p.R271H, *SETX*: p.Y2455C) as variants of uncertain significance, whose pathogenicity needed to be confirmed by further functional studies. Six out of the nine known variants (Additional file 1: Fig. S1a) were identified as pathogenic variants and three as likely pathogenic variants.

**Table 1:** Features of variants identified in this study

Gene	Exon	Nucleotide change	Amino acid change	Mutation type	1000 Genomes	ExAc	In-house data
*SACS*	10	c.7802T > A	p.V2601D	Missense	0	0	0
*SACS*	10	c.7901A > C	p.D2634A	Missense	0	0	0
*SACS*	10	c.8000T > C	p.F2667S	Missense	0	0	0
*SACS*	10	c.8793dupA	p.R2932fs	Insertion	0	0.000008	0
*SACS*	10	c.10685_10689del	p.F3562fs	Deletion	0	0	0
*SACS*	10	c.10938_10941del	p.K3646fs	Deletion	0	0	0
*SACS*	10	c.11274_11276del	p.3758_3759del	Deletion	0	0	0
*SACS*	10	c.11319_11321del	p.3773_3774del	Deletion	0	0	0
*SACS and 5 others*	-	chr13:23490196-24866656 del	–	Deletion	0	0	0
*SYNE1*	5	c.253C > T	p.R85X	Nonsense	0	0.000008	0
*SYNE1*	10	c.909 + 1G > A	–	Splicing	0	0	0
*SYNE1*	27	c.3280A > T	p.K1094X	Nonsense	0	0	0
*SYNE1*	57	c.9158delA	p.E3053fs	Deletion	0	0	0
*SYNE1*	65	c.10435C > T	p.R3479X	Nonsense	0	0	0
*SYNE1*	82	c.15817delG	p.E5273fs	Deletion	0	0	0
*SYNE1*	93	c.17531_17532insTC	p.H5844fs	Insertion	0	0	0
*SYNE1*	114	c.20837delT	p.L6946fs	Deletion	0	0	0
*SYNE1*	131	c.23765 + 1G > A	–	Splicing	0	0	0
*ADCK3*	6	c.812G > A	p.R271H	Missense	0	0.000048	0
*ADCK3*	7	c.901C > T	p.R301W	Missense	0	0.000033	0
*ADCK3*	8	c.960delG	p.L320fs	Deletion	0	0.000009	0
*ADCK3*	10	c.1228C > T	p.R410X	Nonsense	0	0.000017	0
*ADCK3*	15	c.1793G > A	p.R598H	Missense	0	0.000008	0
*ADCK3*	15	c.1844dupG	p.S616fs	Insertion	0	0.000082	0
*SETX*	3	c.128G > A	p.C43Y	Missense	0	0	0
*SETX*	10	c.4818_4821dupAATT	p.A1608fs	Insertion	0	0	0
*SETX*	10	c.5267T > C	p.F1756S	Missense	0	0.000009	0
*SETX*	23	c.7011delT	p.V2337fs	Deletion	0	0	0
*SETX*	26	c.7364A > G	p.Y2455C	Missense	0	0.000008	0
*ANO10*	2	c.132dupA	p.D45fs	Insertion	0.000599	0.000441	0
*SPTBN2*	2	c.73C > T	p.R25C	Missense	0	0	0
*SPTBN2*	6	c.622G > A	p.G208R	Missense	0	0	0
*STUB1*	3	c.433A > C	p.K145Q	Missense	0.000599	0.000702	0
*STUB1*	3	c.433_435delAAG	p.K145del	Deletion	0	0.000017	0
*TTPA*	4	c.553G > T	p.D185Y	Missense	0.000199	0	0
*ATM*	20	c.2922_2923insAA	p.S974fs	Insertion	0	0	0
*ATM*	45	c.6503C > T	p.S2168L	Missense	0.000200	0.000058	0.001754
*KIF1C*	23	c.2663_2664insGAGGT	p.V888fs	Insertion	0	0	0

Gene	Predicted impact	dbSNP	HGMD	Family Segre-gation	ACMG	
					Evidence	Classification
*SACS*	D/P/D	NA	Novel	NA	PM1,PM2,PP3,PP4	LP
*SACS*	D/D/A	NA	Novel	Yes	PM1,PM2,PP1,PP3,PP4	LP
*SACS*	D/D/D	NA	Novel	Yes	PM2,PM3,PP1,PP3,PP4	LP
*SACS*	NA/NA/NA	rs767871841	Novel	Yes	PVS1,PM2,PP1,PP4	P
*SACS*	NA/NA/NA	NA	Novel	Yes	PVS1,PM2,PP1,PP4	P
*SACS*	NA/NA/NA	NA	Novel	Yes	PVS1,PM2,PP1	P
*SACS*	NA/NA/NA	rs1454517884	Known	Yes	PM2,PM4,PP1,PP5	LP
*SACS*	NA/NA/NA	NA	Novel	Yes	PM2,PM4,PP1,PP4	LP
*SACS and 5 others*	NA/NA/NA	NA	Novel	Yes	1A,2A,3A,4A,5A	P
*SYNE1*	NA/NA/A	rs768958602	Novel	Yes	PVS1,PM2,PP1,PP3	P
*SYNE1*	NA/NA/NA	NA	Novel	Yes	PVS1,PM2,PP1,PP3	P
*SYNE1*	NA/NA/A	NA	Novel	Yes	PVS1,PM2,PM3,PP1	P
*SYNE1*	NA/NA/NA	NA	Novel	NA	PVS1,PM2	LP
*SYNE1*	NA/NA/A	NA	Novel	Yes	PVS1,PM2,PP1,PP3	P
*SYNE1*	NA/NA/NA	NA	Novel	Yes	PVS1,PM2,PM3,PP1	P
*SYNE1*	NA/NA/NA	NA	Novel	Yes	PVS1,PM2,PM3,PP1	P
*SYNE1*	NA/NA/NA	NA	Novel	Yes	PVS1,PM2,PP1	P
*SYNE1*	NA/NA/D	NA	Novel	Yes	PVS1,PM2,PP1,PP3	P
*ADCK3*	D/D/D	rs765859566	Novel	Yes	PM2,PP1,PP3	VUS
*ADCK3*	D/D/D	rs140246430	Known	Yes	PS1,PM2,PP1,PP3,PP5	LP
*ADCK3*	NA/NA/NA	rs767164059	Novel	Yes	PVS1,PM2,PP1	P
*ADCK3*	NA/NA/A	rs753254213	Known	Yes	PVS1,PM2,PP1,PP3,PP5	P
*ADCK3*	D/D/D	rs766101783	Novel	Yes	PM2,PM3,PP1,PP3	LP
*ADCK3*	NA/NA/NA	rs863223885	Known	Yes	PVS1,PM2,PP1,PP5	P
*SETX*	D/D/D	NA	Novel	Yes	PM2,PM3,PP1,PP3	LP
*SETX*	NA/NA/NA	NA	Novel	Yes	PVS1,PM2,PP1	P
*SETX*	D/D/D	rs762175796	Known	Yes	PS1,PM2,PM3,PP1,PP3, PP5	P
*SETX*	NA/NA/NA	NA	Novel	Yes	PVS1,PM2,PP1	P
*SETX*	D/D/D	rs778210918	Novel	NA	PM2,PP3,PP4	VUS
*ANO10*	NA/NA/NA	rs540331226	Known	Yes	PVS1,PS1,PM2,PP1,PP5	P
*SPTBN2*	D/D/D	NA	Novel	Yes	PM1,PM2,PP1,PP3	LP
*SPTBN2*	D/D/D	NA	Novel	Yes	PM1,PM2,PP1,PP3	LP
*STUB1*	T/P/D	rs146251364	Known	Yes	PS1,PM1,PM2,PP1,PP5	P
*STUB1*	NA/NA/NA	rs779647632	Known	Yes	PS1,PM2,PM3,PM4,PP1	P
*TTPA*	D/D/D	rs564501015	Novel	Yes	PM1,PM2,PP1,PP3	LP
*ATM*	NA/NA/NA	NA	Novel	Yes	PVS1,PM2,PP1	P
*ATM*	D/D/D	rs200431631	Known	Yes	PM1,PM2,PM3,PP1,PP5	LP
*KIF1C*	NA/NA/NA	NA	Novel	Yes	PVS1,PM2,PP1	P

In-house data: *n* = 2000. The impact of non-synonymous protein-coding region variants was determined using prediction software including SIFT, PolyPhen-2 and Mutation Taste. SIFT results as Tolerated (T) or Deleterious (D). PolyPhen-2 results as Unknown (UN), Benign (B), Possibly Damaging (P) or Probably Damaging (D). Mutation Taste results as Tolerated (T), Disease causing (D) and Disease causing automatic (A). NA, Not available. ACMG, American College of Medical Genetics. ACMG evidence by reference to the ACMG standards and guidelines. ACMG classification including Pathogenic (P), Likely pathogenic (LP) and Uncertain significance (VUS)

Therefore, 25 out of 54 (46.3%) patients were genetically diagnosed with ARCA. Previously, we reported an index patient with AOA type 2 (AOA2) [20] and an index patient with autosomal recessive spinocerebellar ataxia-27 (SCAR27) [21]. Taken together, genetic diagnoses were made for a total of 27 out of 56 (48.2%) ARCA index patients in our center. The most common subtype was ARSACS (22.2%), followed by SCAR8 (18.5%), SCAR9 (18.5%) and AOA2 (14.8%) (Fig. 1c). The clinical features of the patients identified with the genetic variants are listed in Table 2.

**Table 2:** Detailed clinical features of 25 probands with mutation detected by WES

Case no./sex	AAO/DD (years)	Family history	Gene	Disease	Variants	Initial symptom	Ataxia	Dysarthria	Nystagmus	Additional phenotype	Imaging
1/M	1/2	Sporadic	*SACS*	ARSACS	p.K3646fs, chr13:23490196-24866656 del	Gait disturbance	+	−	–	SMN, abnormality of the dentition	Normal
2/M	1/30	Sporadic	*SACS*	ARSACS	p.F2667S, p.F3562fs	Gait disturbance	+	+	H	SMN, spasticity, pes cavus, mild intellectual disability, saccadic pursuit, muscle atrophy and weakness of upper and lower limbs, hearing loss of right ear	Brain and cerebellum atrophy, A*
3/F	1/20	Affected sister	*SACS*	ARSACS	p.R2932fs (Hom)	Gait disturbance	+	+	–	SMN, spasticity, pes cavus	Cerebellum atrophy, A*
4/M	4/22	Sporadic	*SACS*	ARSACS	p.D2634A, p.3773_3774del	Gait disturbance	+	+	H	SMN, spasticity, pes cavus, epilepsy, flexion deformity of fingers, weakness of lower limb	Cerebellum atrophy, craniocerebral dysplasia, A*
5/M	11/26	CP	*SACS*	ARSACS	p.V2601D (Hom)	Gait disturbance	+	+	H	SMN, sensorineural hearing loss of left ear, pes cavus, weakness of lower limb	Cerebellum atrophy, A*
6/M	39/7	CP	*SACS*	ARSACS	p.3758_3759 del (Hom)	Gait disturbance	+	−	–	SMN, weakness of limbs, muscle atrophy of lower limb	Cerebellum atrophy, A*
7/M	9/20	Sporadic	*SYNE1*	SCAR8	p.K1094X, p.E5273fs	Gait disturbance	+	+	H	Psychiatric symptoms, limited abduction and supraduction of eyes, myoclonic jerks	Cerebellar atrophy
8/M	19/20	Affected brother	*SYNE1*	SCAR8	p.E3053fs (Hom)	Gait disturbance	+	−	–	Dysphagia	NA
9/F	21/4	Sporadic	*SYNE1*	SCAR8	p.L6946fs, c.23765 + 1G > A	Gait disturbance	+	+	H	SMN, tremor, dizziness, pes cavus, mental retardation, ankylosing spondylitis	Cerebellar atrophy
10/F	25/0.1	Sporadic	*SYNE1*	SCAR8	c.909 + 1G > A, p.R3479X	Gait disturbance	+	−	H	Sensorineural hearing loss	Cerebellar atrophy
11/F	53/4	Affected brother	*SYNE1*	SCAR8	p.R85X, p.H5844fs	Gait disturbance	+	+	–	Dysphagia, SMN	Cerebellar atrophy
12/M	2/5	Sporadic	*ADCK3*	SCAR9	p.R410X (Hom)	Gait disturbance	+	+	NA	–	Cerebellar atrophy
13/M	9/2	Sporadic	*ADCK3*	SCAR9	p.R271H, p.R301W	Gait disturbance	+	+	NA	Reduced dexterity of hands, cognitive impair	Cerebellar atrophy
14/M	14/3	Sporadic	*ADCK3*	SCAR9	p.R598H, p.S616fs	Hand shake uncontrollably	+	−	NA	Both hands and head shake uncontrollably	Brainstem, cerebellar atrophy
15/M	24/2	Sporadic	*ADCK3*	SCAR9	p.S616fs (Hom)	Hand shake uncontrollably	+	+	–	Both hands and head shake uncontrollably, dysphagia, SMN	Cerebellar atrophy
16/F	32/20	Affected sister	*ADCK3*	SCAR9	p.L320fs (Hom)	Gait disturbance	+	+	H	Right common peroneal neuropathy, cognitive impair, incomplete ptosis of left eyelid	Cerebellar atrophy
17/M	18/2	Sporadic	*SETX*	AOA2	p.C43Y, p.A1608fs	Gait disturbance	+	+	H	Postural tremor of the limbs, tongue muscle tremors, pes cavus, SMN, AFP elevation	Cerebellar atrophy
18/F	18/0.5	Sporadic	*SETX*	AOA2	p.F1756S, p.V2337fs	Gait disturbance	+	+	H	Head shakes uncontrollably, dysphagia, pollakiuria, SMN, AFP elevation	Cerebellar atrophy
19/F	21/10	Sporadic	*SETX*	AOA2	p.Y2455C (Hom)	Diplopia	+	−	H, V	SMN, head shake uncontrollably, reduced dexterity of hands, esotropia of the left eye, SMN, AFP elevation	Cerebellar atrophy
20/F	35/8	CP	*ANO10*	SCAR10	p.D45fs (Hom)	Dizziness	+	+	H	Diplopia, dysphagia, SMN	Cerebellar atrophy
21/M	7/10	Sporadic	*SPTBN2*	SCAR14	p.R25C, p.G208R	Slurred speech	+	+	–	Sensorineural hearing loss, intellectual disability	Normal
22/F	21/11	Sporadic	*STUB1*	SCAR16	p.K145Q, p.K145del	Psychiatric symptoms	+	+	H	Depression	Cerebellar atrophy
23/F	8/4	Sporadic	*TTPA*	AVED	p.D185Y (Hom)	Gait disturbance	+	+	H	Both hands and head shake uncontrollably, SMN	Cerebellar atrophy
24/F	12/5	Affected brother	*ATM*	AT	p.S974fs, p.S2168L	Psychiatric symptoms	+	+	–	Dizziness, head shake uncontrollably, autonomic dysfunction, scoliosis, pes cavus	NA
25/M	8/8	Sporadic	*KIF1C*	SPAX2	p.G885fs (Hom)	Paroxysmal headaches	+	+	–	Limbs shake uncontrollably, voice tremble, abnormal EEG	Myelin dysplastic

*F* female, *M* male, *AAO* age at onset, *DD* disease duration, *Hom* homozygous, *H* horizontal, *CP* consanguineous parents, *V* vertical, *NA* not available, *SMN* sensorimotor neuropathy, *EEG* electroencephalogram

A*: Thinning of the corpus callosum, bulky pons, bilateral pontine linear hypointense lesion and hyperintensities around the thalami

### Identification of a de novo microdeletion and clinical features of patients with *SACS* mutations

**Fig. 2:**
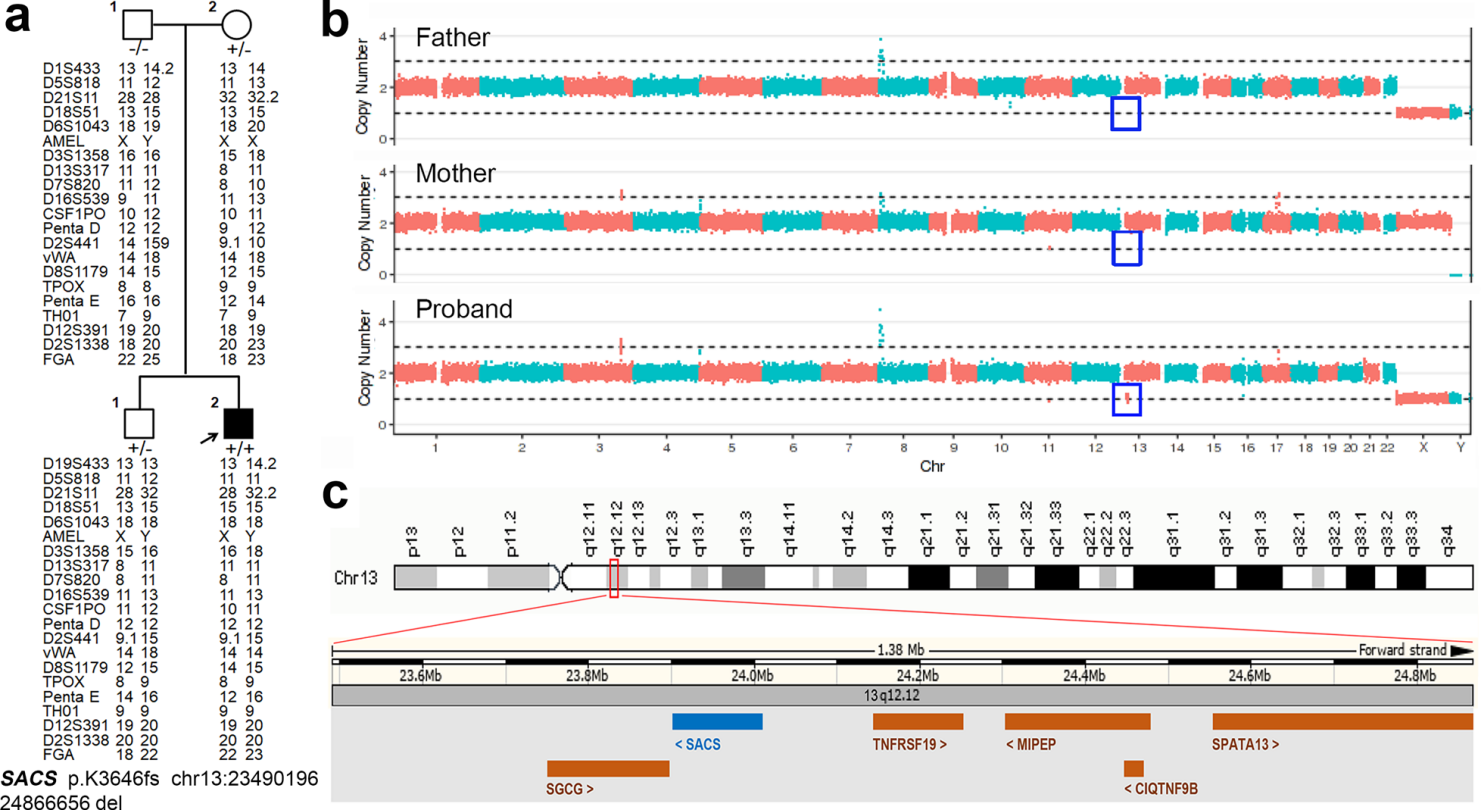
A de novo large deletion in a patient with ARSACS. **a** The pedigree of case 1 shows segregation of p.K3646fs in *SACS* and chr13:23490196-24866656del. The analysis of the repeat numbers of 21 core short tandem repeat loci in the four participants showed that the probability of the patient being the alleged parents’ biological son was 99.99%. Open symbol: unaffected; filled symbol: affected; square: male; circle: female; arrow: proband of the family. Symbol with “+/+” indicates patient. Symbols with “+/−” indicate mutation carrier. **b** CNVseq of the proband and his parents showed that chr13:23490196-24866656del was a de novo mutation, as indicated by the blue box. **c** Schematic diagram of the known genes in this deletion region (reference human Genome Build GRCh37, UCSC Assembly hg19)

After WES analysis, a novel homozygous mutation (p.K3646fs) was identified in case 1 without family history (Table 2). However, the heterozygous p.K3646fs was only confirmed in his mother and brother but not in his father after family segregation. The parent–child relationship was also established by parenthood analysis (Fig. 2a). Therefore, CNV analysis with ExomeDepth was conducted in the proband and a large deletion was detected in chromosome 13. Then, a trio-copy number variation sequencing (CNVseq) was performed and identified a 1.38 Mb deletion (chr13:23490196-24866656del) in the proband, but the corresponding chromosome in the parents was normal, which means it was a de novo large deletion (Fig. 2b). The chr13:23490196-24866656 in Genome browser includes the entire *SACS* gene and 5 other genes (Fig. 2c). Thus, a total of nine mutations including eight novel ones in *SACS* were identified in six patients (Table 2).

**Fig. 3:**
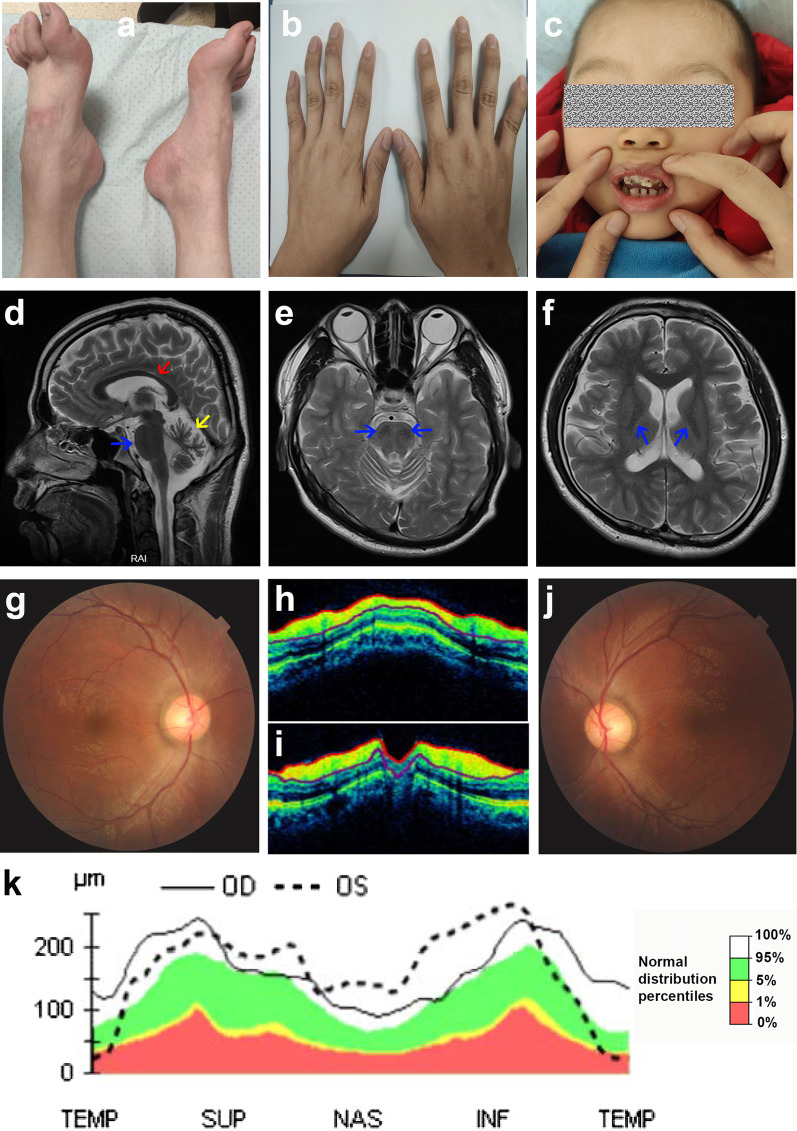
Clinical features of patients with ARSACS. **a**–**c** The special phenotypes of ARSACS: pes cavus (**a**), flexion deformity of fingers in case 4 (**b**) and abnormality of dentition in case 1 (**c**). **d**–**f** Classic brain magnetic resonance images in case 2. **d** Sagittal T2 sequence shows thinning of the corpus callosum (red arrow), superior vermian atrophy (yellow arrow), and bulky pons (blue arrow); **e** axial T2 shows bilateral pontine linear hypointense lesions (arrow); **f** axial T2 shows hyperintensities around the thalamus (arrows). **g**–**k** Typical retinal findings in case 4: Fundus photographs of the right (**g**) and left eyes (**j**) show yellow streaks of hypermyelinated fibers radiating from the edges of the optic disc; ocular coherence tomography imaging of the right (**h**) and left eyes (**i**) show thickened retinal nerve fiber layer (RNFL) (the yellow areas); **k** statistical graph of OCT showing thickening of the RNFL. The black line indicates oculus dexter (OD), that is, the right eye; the dotted line indicates oculus sinister (OS), the left eye. The green band represents the 5%–95% range of the normative data. (Quadrants: *TEMP* temporal, *SUP* superior, *NAS* nasal, *INF* inferior)

Of the six patients, the mean age of disease onset was 9.5 years (1–39 years), and onset with gait disturbance was all accompanied by sensorimotor neuropathy. Weakness of limbs was present in 4 cases, skeletal abnormality in 5 cases (pes cavus in 4 cases [Fig. 3a] with 1 accompanied by flexion deformity of fingers [Fig. 3b], dental abnormalities in 1 case [Fig. 3c]), spasticity in 3 cases, listening loss in 2 cases, and mental retardation in 1 case. Radiological evaluation of the 3-year-old patient (case 1) showed a normal contract, whereas the other 5 cases showed cerebellum atrophy, thinning of the corpus callosum, bulky pons, bilateral pontine linear hypointense lesions and hyperintensities around the thalamus (Fig. 3d–f), and one had craniocerebral dysplasia (case 4). A characteristic retinal finding of case 2 and case 4 was the presence of yellow streaks of hypermyelinated fibers radiating from the edges of the optic disc and retinal nerve fiber hypertrophy, as demonstrated on ocular coherence tomography (Fig. 3g–k).

### Characteristics of patients with *SYNE1* mutations

Among the nine novel *SYNE1* pathogenic variants found in five cases, four were frameshift, three were nonsense and two were splicing variants. The onset age was before 25 years in four patients and at 53 years (late onset) in one patient. All of the five cases had onset with gait disturbance, and three of them had dysarthria and horizontal nystagmus. Case 7 had the earliest onset with most complex phenotypes including psychiatric symptoms, external ophthalmoplegia and myoclonic jerks (Additional file 2: Video S1). Case 8 with a homozygous mutation (p.E3053fs) appeared to be pure cerebellar ataxia. Both case 9 and case 11 had sensorimotor neuropathy, and case 9 was also accompanied by tremor, dizziness and pes cavus. Case 10 had a 15-year history of sensorineural hearing loss and mild ataxia. She also exhibited two pathogenic mutations (Additional file 1: Fig. S1b) including c.6149-3T > G and c.1898dupA (p.E633fs) in *PTPRQ* (NM_001145026.1). After pedigree verification, her brother affected by impaired hearing but without ataxia, was found to carry these two mutations in *PTPRQ* but only one heterozygous mutation in *SYNE1*, and all mutations in *SYNE1* and *PTPRQ* were derived independently from two parents with normal phenotypes (Additional file 1: Fig. S1c). Owing to the lack of magnetic resonance imaging report of case 8, the remaining four probands were estimated to have a varied degree of cerebellar atrophy.

### Identification of a rare UPD and clinical features of patients with *ADCK3* mutations

**Fig. 4:**
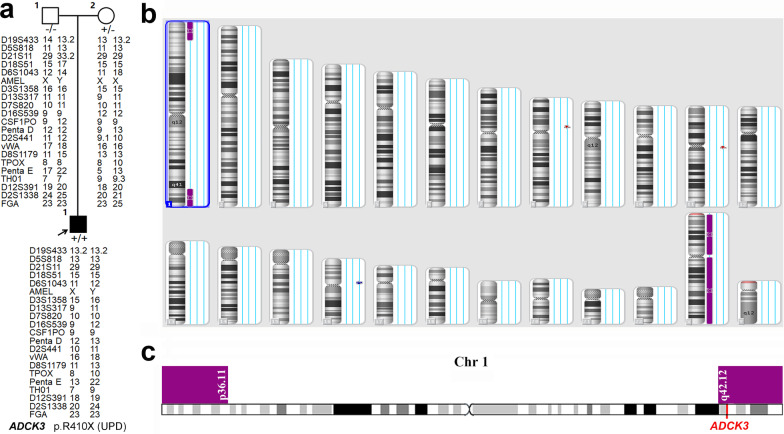
A maternal uniparental disomy in a patient with SCAR9. **a** The pedigree of case 12 shows the segregation of p.R410X in *ADCK3*. The analysis of the repeat numbers of 21 core short tandem repeat loci in the three participants showed that the probability of the patient being the alleged parents’ biological son was 99.99%. **b** Uniparental disomy of chromosomes 1p and 1q detected by Affymetrix CytoScan® Dx Assay in the patient. **c** Schematic diagram of *ADCK3* in this UPD region (1pterp36.11 and 1q42.12qter; reference human Genome Build GRCh37, UCSC Assembly hg19)

After WES analysis, six *ADCK3* mutations including three novel ones (p.R271H, p.L320fs and p.R598H) were identified in four males with no family history of disease and one female with an affected sister. The homozygous variant (p.R410X) within *ADCK3* was identified in case 12 (Table 2). However, the heterozygous p.R410X was only confirmed in his mother but not in his father, and the parental analysis was normal (Fig. 4a). The CNV analysis of the index patient was normal, too. Thus, UPD was considered in this situation. The results of Affymetrix CytoScan® Dx Assay analyses were as follows: arr [hg19] 1pterp36.11 (888,658-25,445,510) hmz; arr [hg19] 1q42.12qter (225,105,798-249,198,164) hmz, which suggested that the index patient had maternal UPD for a segment of chromosome 1 (Fig. 4b), which included the whole *ADCK3* gene (Fig. 4c).

The mean age at onset was 16.2 years (range 2–32 years). All patients showed ataxia signs, and four also displayed dysarthria. Cases 12, 13 and 16 had onset with gait disturbance, case 12 displayed pure ataxia, while cases 13 and 16 had additional cognitive impairment. Cases 14 and 15 presented onset with uncontrollable hand shaking, followed later by head shaking. Both were identified to have the p.S616fs mutation, but the mutation was heterozygous in case 14 and homozygous in case 15. Case 15 had additional dysphagia and sensorimotor neuropathy. Magnetic resonance imaging revealed that all five patients had cerebellar atrophy, and one also had atrophy of the brainstem. Case 15 was of the Dong ethnic minority and had started supplementation with 300 mg/day of CoQ10. At 1-year follow-up, he showed clinical improvement, which was more evident in the tremor and gait items. His total Scale for the Assessment and Rating of Ataxia scores were 10 at baseline and 4.5 after 1 year; similarly, his International Cooperative Ataxia Rating Scale score decreased from 21 to 9, and his non-ataxia score decreased from 4 to 1 (Additional file 3: Table S1). A homozygous mutation was identified by Sanger sequencing in his 24-year-old sister, who had no clinical symptoms yet.

## Discussion

To date, few studies have been conducted to investigate all causative genes and the clinical features of ARCA in the Chinese population. In this study, the prevalence of ARCA was systematically investigated in 54 unrelated autosomal recessive/sporadic ataxia patients by WES analysis and CNV calling, which is the largest cohort in China to date. Thirty-eight mutations, including 29 novel mutations in 10 genes related to ARCA, were identified in 25 unrelated patients. Among them, the de novo microdeletion in *SACS* was reported for the first time in the Chinese population and the UPD of *ADCK3* was reported for the first time worldwide.

In this study, the diagnostic yield was 46.3% (25 of 54 patients). This rate is at a relatively high level compared to those previously reported using exome sequencing [5, 22–27]. Marelli et al. also performed a mini-exome and read-depth-based CNV analysis in 33 ataxic patients and identified pathogenic variants in 14 cases (42%) including CNV in 2 patients [28]. The differences in diagnostic rate were most likely due to the differences in source populations (ethnic and geographic origin), sample size, the inclusion criteria used and study methodology. One of the major highlights of the present study is the combination of WES and CNV analysis, and the results truly demonstrate that structural variation might not be extremely rare in ARCA. With the development of high throughput sequencing technology and bioinformatics algorithms, many established approaches for CNV calling in WES data have been available and easily usable for biologists and geneticists to test structural variations, such as ExomeDepth, CovCopCan, IonCopy, DeviCNV, and Cov’Cop [29]. Thus, we suggest that tools for detecting structural variations such as CNVs should be used routinely for NGS data analysis in order to increase the rate of positive diagnosis.

In this study, 12 autosomal recessive ataxia and 42 sporadic ataxia families were included. Mutations in ARCA causative genes were identified in eight autosomal recessive ataxia and 17 sporadic ataxia families. Combined with our previous studies, the number of ARCA families with definite diagnosis in our center was 27. Further, we demonstrate that ARSACS (gene: *SACS*; *n* = 6, 22.2%), SCAR8 (gene: *SYNE1*; *n* = 5, 18.5%), SCAR9 (gene: *ADCK3*; *n* = 5, 18.5%) and AOA2 (gene: *SETX*; *n* = 4, 14.8%) are the most common recessive ataxia subtypes in the Chinese population. In a previously reported Chinese ARCA cohort, the ARCA-causing genes were identified in 19 out of 26 probands, including AOA2 (*n* = 4, 15.4%), Niemann-Pick disease (*n* = 3, 11.5%), one ARSACS and one SCAR8 [27]. *SACS* and *SYNE1* mutations have been observed mainly in Quebec and Canada, where ARSACS and SCAR8 are the second and third most common hereditary ataxia, respectively [7]. FRDA has been reported as the most frequent ARCA in Caucasians but is much rare in Chinese [30], thus the identification of dynamic mutations about FRDA was not undertaken in our study. Although the aetiology of ARCA in Chinese is different from the reported patterns in Caucasians presumably due to the different genetic backgrounds and ethnicities, there are still some similarities which may contribute to a better understanding of the epidemiology and mechanism of ARCA.

In total, 14 ARSACS patients from 10 families have been reported in China (Additional file 4: Table S2), including the 6 probands in this study, confirming that nonsense or frameshift mutations in *SACS* are the most common genetic cause in Chinese patients. In our study, a majority of those patients who had at least one truncation variant appeared to have a typical ARSACS clinical presentation with childhood onset of symptoms. But one patient who harbored a homozygous non-frameshift deletion variant (p.3758_3759del), exhibited an atypical disease presentation with an absence of spasticity or pyramidal signs and onset in adulthood, and this same variant has been reported in two heterozygous patients, both with early onset (1 and 13 years old) and typical triad symptoms [31, 32]. Thus, whether the truncation variant in *SACS* is linked to a typical clinical manifestation of ARSACS is an issue that requires further exploration. Even this de novo large deletion containing *SACS* was the second report in the world [33], the CNV in *SACS* has already been reported in many populations including Belgian, French, Italian, Canadian, German and Chinese [33–39]. Therefore, presence of CNV must be considered if no or only one heterozygous mutation had been identified in those patients with an ARSACS phenotype suggested by means of clinical presumption or auxiliary examination.

Defects in *SYNE1* are associated with adult-onset, slowly progressive, relatively pure cerebellar ataxia with only a few extracerebellar symptoms (SCAR8), and almost all reported variants that cause this phenotype are protein truncations [7]. Previous studies showed that *SYNE1* ataxia accounted for 5.3% (23/434), 6% (7/116), and 10.26% (4/39) of recessive and sporadic ataxia patients in two European combined cohorts and one Brazilian cohort [7, 40, 41]. In the present study, 9.3% (5/54) of ataxia patients had biallelic truncating variants in *SYNE1*, demonstrating that SCAR8 is also a common subtype of recessive ataxia in China. Two SCAR8 patients (cases 8 and 10) both had symptoms of pure cerebellar ataxia, while the hearing loss of case 10 was caused by mutations in *PTPRQ*. Moreover, the remaining three patients exhibited variable additional extracerebellar neurological symptoms (peripheral polyneuropathy, mental retardation, dizziness, pes cavus, external ophthalmoplegia, myoclonic jerks) and non-neurologic dysfunctions (psychiatric symptoms). The reported nine SCAR8 Chinese patients included three presenting with pure cerebellar ataxia and six presenting with variable ataxia syndrome [27, 42, 43]. Thus, in the Chinese population, pure cerebellar ataxia only accounted for 35.7% (5/14) of SCAR8 cases, while the other 64.3% (9/14) of patients showed complex ataxia phenotypes with a wide range of noncerebellar abnormalities. This further supports the concept that *SYNE1* ataxia is a multisystemic neurodegenerative disease, as proposed by Synofzik et al. [7].

A total of 65 pathogenic mutations in *ADCK3* have been reported around the world (HGMD, Professional 2021.1), and SCAR9 has also been reported as a common subtype of recessive ataxia [26]. However, cases of SCAR9 were rarely reported among the Chinese population before [44]. Here, we report for the first time that SCAR9 also had a high frequency in China. The homozygous p.S616fs in *ADCK3* has been reported in two siblings from a consanguineous family of Pakistani origin, and both siblings presented with cerebellar ataxia, myoclonus, tremor and dysarthria at age of 10 and 14, respectively [45]. However, in our study, two patients harboring p.S616fs in heterozygous or homozygous form both presented with prominent tremor and mild ataxia symptoms, but onset at adolescence and adulthood, respectively. Moreover, the homozygous patient presented with additional dysphagia and peripheral neuropathy, and his sister was identified to have the mutation but was still asymptomatic at the age of 24. Thus, the clinical presentation of SCAR9 may be highly variable, even in patients with the same mutation from one family. Our study also proved that supplementation with COQ10 is significantly helpful for SCAR9 patients, even though this therapy has different curative effects in several studies [45–47]. The UPD of *ADCK3* identified in our study is the first report worldwide, which not only enriches the genotypic spectrum of SCAR9, but also emphasizes the importance of a detailed analysis of family segregation.

The absence of diagnosis for 29 patients in the cohort may be explained by the following reasons. First, it is difficult for exome capture to fully cover all coding regions of the genome, especially regions rich with GC. Second, large genomic rearrangements and trinucleotide expansions cannot be reliably detected from exome-capture data, even though there are some CNV-detecting tools developed, and based on the read-depth of NGS data, such as ExomeDepth used in this study, we cannot easily detect inversion or translocation [34]. Third, it is also likely that some causal variants are outside the coding regions and adjacent splice sites [48]. Finally, insights into the functional consequences of the variants are missing, as some synonymous mutations may be causative, too [49]. Some of these issues can be addressed by whole-genome sequencing, which is, however, expensive and needs complex bioinformatics analyses. In addition, mutations in yet unknown genes of ARCA may play a key role in these unclear disorders.

## Conclusions

In summary, this is so far the largest WES analysis and CNV calling study to explore the genetic background and describe clinical characteristics of ARCA in a Chinese population. We identified 38 mutations including 29 novel ones in 25 unrelated Chinese ARCA patients. We also reported for the first time the UPD of *ADCK3* in the world and the de novo microdeletion of *SACS* in China. Our results expand the genetic spectrum and clinical profiles of ARCA patients, demonstrate the high efficiency and reliability of WES combined with CNV analysis in diagnosing suspected ARCA, and emphasize the importance of complete bioinformatics analysis of WES data in making an accurate diagnosis. Further functional studies will help to determine the pathogenicity of novel variants and to better understand the pathogenetic mechanisms of these complicated diseases.

## Supplementary Information

**Additional file 1: Figure S1.** Reported mutations identified in our ARCA patients. **a** Sequencing chromatograms of 9 reported ARCA-related mutations. The upper sequence in each frame represents the normal sequence, whereas the lower one represents the variant. **b** Sequencing chromatograms of *PTPRQ* mutations in case 10. **c** The pedigree of case 10 with ataxia and hearing loss. Open symbol: unaffected; filled symbol with black or red: affected with ataxia or hearing loss; square: male; circle: female. Genotype data are shown underneath the symbols. Arrowhead: proband of the family.

**Additional file 2: Video S1.** Myoclonic jerks of face in case 7 with *SYNE1* mutations.

**Additional file 3: Table S1.** The scale score follow-up of case 15 with ADCK3 mutation after supplementation with COQ10.

**Additional file 4: Table S2.** Clinical features of ARSACS patients in China.

## Data Availability

The data presented in this study are available on request from the corresponding author. The data are not publicly available due to privacy reasons.

## Abbreviations

ACMG: American College of Medical Genetics and Genomics
AOA: Ataxia with oculomotor apraxia
ARCA: Autosomal recessive cerebellar ataxia
ARSACS: Autosomal-recessive spastic ataxia of Charlevoix-Saguenay
CNV: Copy number variation
FRDA: Friedreich’s ataxia
HCA: Hereditary cerebellar ataxia
HGMD: Human gene mutation database
SNV: Single-nucleotide variant
*SYNE1*: Spectrin repeat-containing nuclear envelope protein
WES: Whole-exome sequencing
UPD: Uniparental disomy
