# Supplementary figures and images for: Genetic spectrum and clinical features in a cohort of Chinese patients with autosomal recessive cerebellar ataxias
Source: Transl Neurodegener. 2021 Oct 18;10:40. doi: 10.1186/s40035-021-00264-z (PMC8522248; doi:10.1186/s40035-021-00264-z)

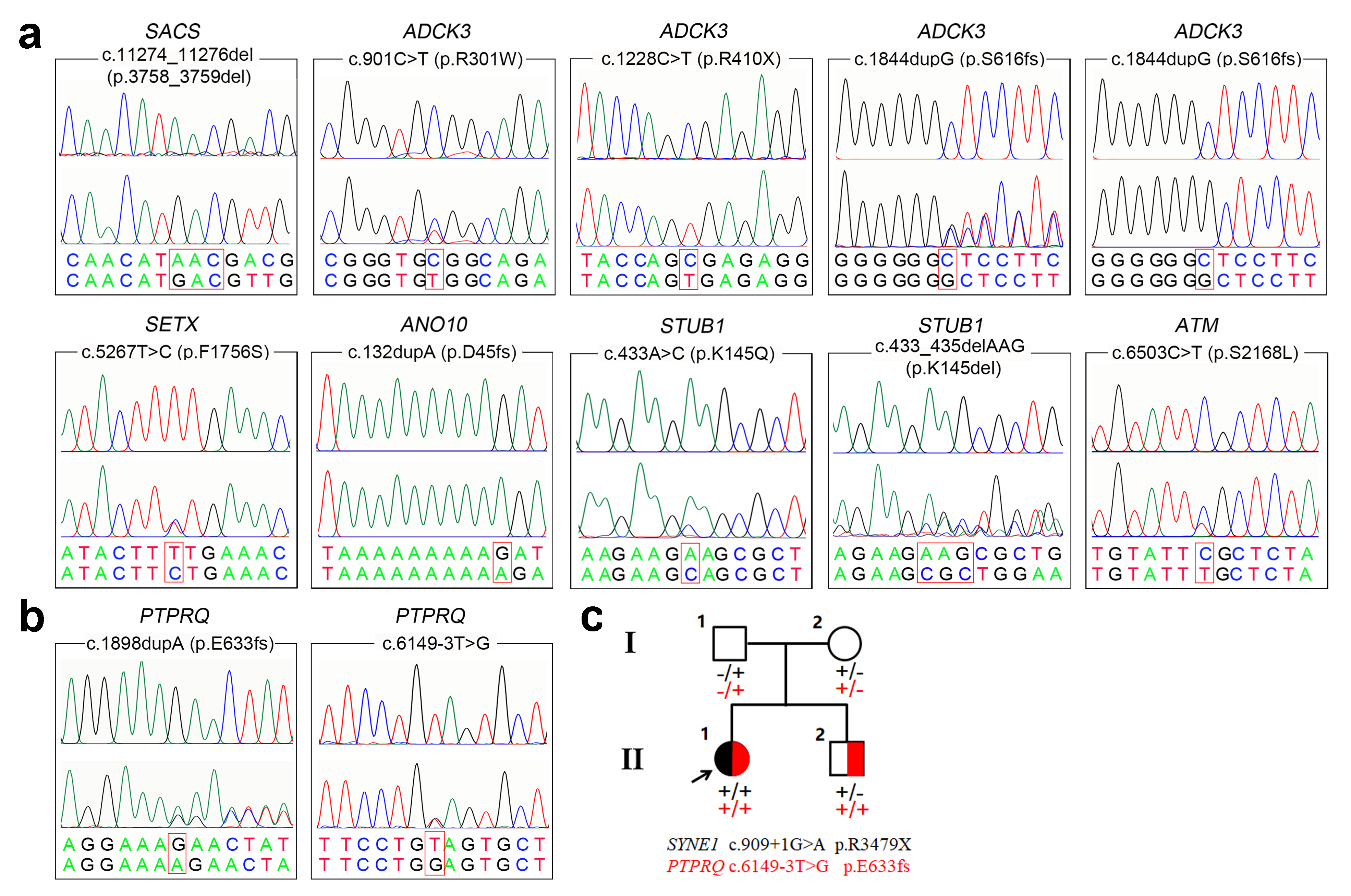

Supplement: Supplementary file 1 — Additional file 1: Figure S1. Reported mutations identified in our ARCA patients. a Sequencing chromatograms of 9 reported ARCA-related mutations. The upper sequence in each frame represents the normal sequence, whereas the lower one represents the variant. b Sequencing chromatograms of PTPRQ mutations in case 10. c The pedigree of case 10 with ataxia and hearing loss. Open symbol: unaffected; filled symbol with black or red: affected with ataxia or hearing loss; square: male; circle: female. Genotype data are shown underneath the symbols. Arrowhead: proband of the family. [file 40035_2021_264_MOESM1_ESM.tif]
